# Metastasis: Other Side of the Coin

**DOI:** 10.3389/fonc.2015.00163

**Published:** 2015-08-04

**Authors:** Aftab Ahmad, Shoji Komai

**Affiliations:** ^1^National Academy of Young Scientists (NAYS) Pakistan, Lahore, Pakistan; ^2^Graduate School of Biological Sciences, Nara Institute of Science and Technology (NAIST), Ikoma, Japan

**Keywords:** regression, immune system, nervous system, dilution, proliferation

Metastasis is a leading cause of poor prognosis and cancer death worldwide and according to reports, metastasis results in almost 90% cancer-related deaths (1). The early detection of cancer and better understanding of cancer metastasis can be incredibly helpful in management of disease (2).

Recently, there is a great advancement in metastasis-related research but yet prognosis remains extremely poor. Metastasis is a very complex process, which is mainly through four steps: (i) departure of cells from the primary tumor, (ii) movement and survival of cancerous cells in circulator system, (iii) attachment of cells with the target organ through breaching of endothelial and basement membrane, and (iv) formation of metastatic cells colony in target organ (3). There are many players who are involved in these steps including nervous system, microenvironment, inflammation, immune system, and bone marrow (4–6). Among all these factors, nervous and immune system play an important role, directly or indirectly (7).

Angiogenesis is very important for metastasis (8) and for angiogenesis; vascular endothelial growth factor (VEGF) plays an important role. According to the reports, the level of VEGF increases in chronic stress, which result in vascularization and lead to metastasis (9, 10). In addition, under chronic stress, the sympathetic nervous system (SNS) also activated, which release neurotransmitters and these neurotransmitters, such as norephedrine, dopamine, and bradykinin, induce or suppress VEGF expression (11, 12).

Other neuron-related factors also play an important role in tumor angiogenesis like neuropilins and semaphorins. These factors can promote or inhibit the angiogenesis process (13–15). Moreover, calcitonin gene-related peptide (CGRP), which is a neuropeptide, can also facilitate angiogenesis in tumor (16). Another neuropeptide, neuropeptide Y (NPY), is also involved in angiogenesis by regulating the expression of VEGF (17). Overall, nervous system modulates angiogenesis, which further lead to metastasis.

According to a recent study by Magnon et al. (18), tumor sections of mouse contained tumor infiltrating sympathetic and parasympathetic nerves and along with mature there were also some newly formed nerves. In the same study, when sympathetic nerve were ablated surgically or chemically, the growth of tumor was prevented in mice and similar results were also obtained when same nerve were ablated in genetic model of prostate cancer. In the later case, only cancer was prevented in young mice but no effect was observed in adults, which indicate that SNS is important for early tumorigenesis (18). The mechanism of tumor growth by SNS was also revealed in this study and that was through β2-adrenergic receptor (ADRB2). ADRB2 has been shown to be expressed on tumor cells, while SNS transmits stress signals, which activate these receptors and result in tumor growth.

Some recent investigations have challenged the canonical theory of tumor metastasis and according to new theory, the cancerous cells have an innate property of metastasis and malignant cells can disseminate and release into circulation much earlier than previously anticipated (19). In addition, the non-neoplastic host cells in tumor microenvironment including neuronal cells also play a significant role in regulation of metastasis (18).

Metastasis is always said to be a bad process and usually it is considered as “death statement” for patient but can it be beneficial? According to a study by Peeters et al. (20), primary tumor may inhibit the growth of metastasized tumor, as the vascular density in human liver metastatic cancer increased after primary tumor resection from colon (20). According to this study, primary tumor blocks the growth of metastasized tumor by blocking angiogenesis process. In this way, the stress at primary tumor place is reduced by release of cells and primary tumor further helps in blocking the growth of metastatic tumor.

There are several cases where there was spontaneous regression of primary tumors and especially renal cell carcinoma (RCC). Like metastasis, the process of regression is also very complex and many genetics, epigenetic, immunological, and neurological factors are involved, which act through apoptosis, immune system activation, tumor microenvironment, inhibition of metalloproteinases, lack of angiogenesis, and absence of specific proteins. All these factors act together and result in tumor regression (21, 22). In addition, there are also several documented cases of spontaneous regression of metastatic tumor, but what mechanism is involved in this regression is still unknown and needs further investigations (23–25).

According to different reports, all type of tumors can regress spontaneously, although, some cancers regress more frequently than others. In addition, not only malignant but regression was also observed in benign tumors [Sante Basso (26)]. Moreover, the dilution in numbers of cancer cells in the blood also stimulates the immune system and result in regression. It was observed that metastasis was less frequent in renal carcinoma patients who undergo hemodialysis, which indicates that, the number of cancer cells reduced due to their blockage using dialytic membrane. Cancer cells are slowly released in circulation from primary tumor, so more accessible to immune cells; and hence, immune system recognize these cells in better way and prepare the body to fight against cancer, which could be hard in primary solid tumor [S Basso (27); Sante Basso (26)].

The primary tumor normally escape the anti-tumor response of the immune system and many factors play an important role for this escape, especially tumor microenvironment and immune system exclusion and ignorance (28, 29). It indicates that the access of immune cells to primary tumor is less. In addition, if the patient are administered with autologous cancer cells, they enhance the anti-tumor immunity in patients, especially when the cells were engineered to secrete granulocyte-macrophage colony stimulating factor (GM-CSF), which indicate that circulating cancer cells are better recognized by immune cells and they mount anti-tumor response (30, 31), so metastasis make the tumor cells more accessible to immune cells.

According to reports, the process of regression is more common in metastasized tumor compared to primary tumors [Sante Basso (26)]. When cancer cells move to new location, the microenvironment at new location also affect the cancer cells and block its proliferation. In addition, microenvironment also lock the cancer cells, which reduce their number in circulation and so help in mounting the immune response due to dilution factor of cancerous cells. The cells releases from primary tumor are also not always form a tumor at new location. Moreover, after release from the primary tumor, certain tumor cells end up into lymph nodes, which are highly active sites of immune system, so these cancer cells are not only blocked but also greatly stimulate the immune system, which result in spontaneous regression of cancer. Several such cases of spontaneous regression of lymph node metastasis have been reported (32). How metastasis is beneficial for the body is simply illustrated in Figure 1.

**Figure 1:**
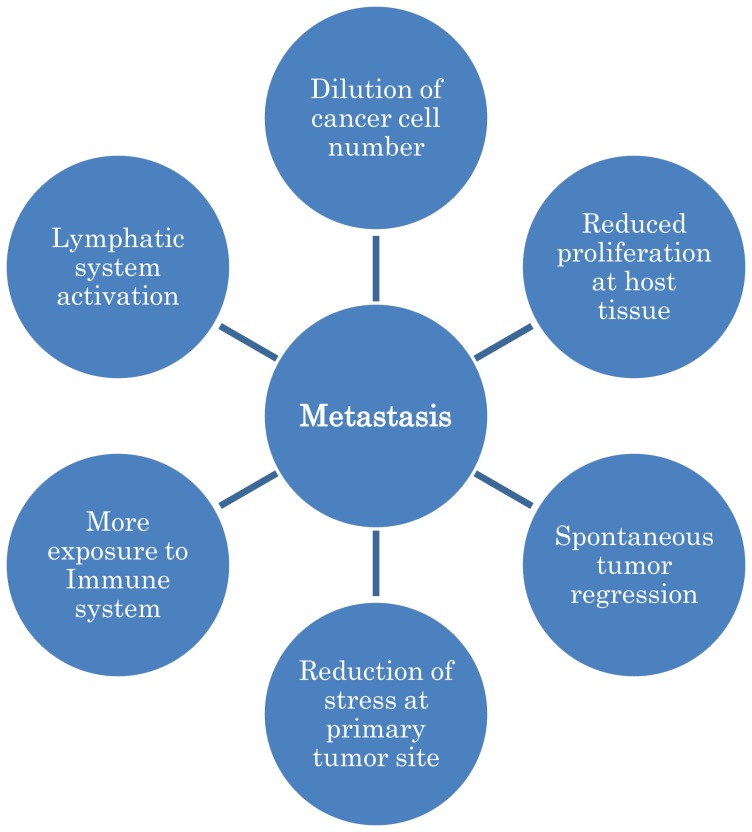
**A simple model to illustrate that how metastasis is helpful for the body**.

On the basis of above discussion, it can be concluded that although metastasis is not good for treatment of cancer, as it is hard to treat cancer when it is not localized but there is also need to re-think about metastasis process, as it is a body’s response to target the diseases and mount an immune response against cancerous cells. The nervous system modulate and facilitate metastasis to reduce the stress of cancer mass at its primary location and better expose cancer cells, so the immune system could recognize them and target these cells not only at its primary location but also at metastatic locations. Just like the old theory of release of cancer cells from primary tumor is changed, we have to re-think about metastatic process as metastasis is also helpful for the body in number of ways (Figure 1). The new theory can also give us many potential new targets and ways to target cancer.

## Conflict of Interest Statement

The authors declare that the research was conducted in the absence of any commercial or financial relationships that could be construed as a potential conflict of interest.
